# INTRODUCING IJD SYMPOSIUM

**DOI:** 10.4103/0019-5154.53174

**Published:** 2009

**Authors:** Sandipan Dhar

**Affiliations:** *Editor, IJD E-mail: drsandipan@gmail.com*

From this issue we are starting a new section called the ‘IJD Symposium,’ which we hope to feature regularly in our forthcoming issues, on a wide range of subjects. Our objective is to showcase articles on issues of particular relevance to students, clinicians, and researchers of dermatology in this subcontinent, although we shall welcome articles having a still wider appeal. The contributors to the sections will have a good number of original research works and will share their vast experience with the readers. We have seen to it that all the topics of the symposium are related to issues that are practical and useful to not only academicians and researchers, but also to each and every practicing dermatologist.

The topic of the first IJD symposium is Vitiligo Surgery.

Dr. Koushik Lahiri, the executive editor of the journal and a renowned worker in the field, has conceived the specific subsections, assigned experienced authors, and compiled the section focused on the topic in a planned and structured manner as the Section Editor. The assigned authors for this section Dr. Mysore Venkataram and Dr. T. Salim, and Dr. Niti Khunger, Dr. Sushruta Das Kathuria and Dr. V. Ramesh have enriched the subject with their vast experience on various techniques of tissue grafts and cellular grafts in vitiligo.

I am sure the readers will find the section quite useful.

